# Transjugular diagnostic procedures in hepatology: Indications, techniques and interpretation^☆^^☆☆^

**DOI:** 10.1016/j.jhepr.2025.101437

**Published:** 2025-04-29

**Authors:** Dominik Bettinger, Annalisa Berzigotti, Mattias Mandorfer, Cristina Ripoll, Christian Labenz, Eugen Zizer, Tony Bruns, Andrea De Gottardi, Johannes Emrich, Cornelius Engelmann, Benjamin Maasoumy, Arnulf Ferlitsch, Valentin Fuhrmann, Jan Hinrichs, Christian Jansen, Karoline Lackner, Robert Matzberger, Carsten Meyer, Behrang Mozayani, Michael Praktiknjo, Philipp A. Reuken, Michael Schultheiss, Alexander Zipprich, Christian M. Lange, Roman Kloeckner, Christoph Sarrazin, Jonel Trebicka, Thomas Reiberger, Jaume Bosch, Matthias M. Dollinger, German (D) – Austrian (A) – Swiss (CH) portal hypertension (DACH-PH) consortium

**Affiliations:** 1 Department of Medicine II, Medical Center University of Freiburg, Faculty of Medicine, University of Freiburg, Hugstetter Str. 55, D-79106 Freiburg, Germany; 2 Department of Visceral Surgery and Medicine, Inselspital, Bern University Hospital, University of Bern, Switzerland; 3 Division of Gastroenterology and Hepatology, Medical University of Vienna, Vienna, Austria, Vienna Hepatic Hemodynamic Lab, Medical University of Vienna, Vienna, Austria, Department of Medicine III and Clinical Research Group Mechanisms in Portal Hypertension, Medical University of Vienna, Vienna, Austria; 4 Internal Medicine IV, Department for Gastroenterology, Hepatology, Interdisciplinary Endoscopy and Infectious Diseases, Jena University Hospital, Friedrich-Schiller University, Jena 07747, Germany; 5 Department of Internal Medicine, University Medical Center of the Johannes Gutenberg-University Mainz, Mainz, Germany; 6 Kreiskliniken Günzburg-Krumbach, Lindenallee 1, 89312 Günzburg, Germany; 7 Department of Internal Medicine III, University Hospital RWTH Aachen, Aachen, Germany; 8 Luzerner Kantonsspital, Lucerne, Switzerland; 9 Medizinische Klinik II, St. Josefs-Hospital, Wiesbaden, Germany; 10 Medical Department, Division of Gastroenterology and Hepatology, Campus Virchow-Klinikum, Charité-Universitätsmedizin Berlin, Corporate Member of Freie Universität Berlin and Humboldt-Universität zu Berlin, Berlin, Germany; 11 Department of Gastroenterology, Hepatology, Infectious Diseases and Endocrinology, Hannover Medical School, Hannover, Germany; 12 Department of Internal Medicine I, St John of God Hospital, Vienna, Austria; 13 Klinik für Innere Medizin, Heilig Geist Krankenhaus Köln, Graseggerstraße 105, 50737, Köln, Germany; 14 St. Bernward Krankenhaus, Klinik für diagnostische und interventionelle Radiologie und Neuroradiologie, Hildesheim, Germany; 15 Department of Internal Medicine I, University Hospital Bonn, Bonn, Germany; 16 Diagnostic and Research Institute of Pathology, Medical University of Graz, 8010, Graz, Austria; 17 Department of Medicine I (Gastroenterology, Hepatology, Diabetology & Nephrology), Klinikum Landshut, Landshut, Germany; 18 Department of Radiology, University Hospital, University Bonn, Bonn, Germany; 19 Department of Pathology, Medical University of Vienna, Vienna, Austria; 20 Department of Medicine B, Gastroenterology, Hepatology, Endocrinology, Infectious Diseases, Universitätsklinikum Münster, Münster, Germany; 21 Department of Internal Medicine II, LMU University Hospital Munich, Munich, Germany; 22 Institute for Interventional Radiology, University Hospital Schleswig-Holstein Campus Luebeck, Lübeck, Germany

**Keywords:** Cirrhosis, porto-sinusoidal vascular disorder, PSVD, portal hypertension, hepatic venous pressure gradient, HVPG, transjugular liver biopsy, minimally invasive

## Abstract

Measurement of the hepatic venous pressure gradient (HVPG) and transjugular liver biopsy have emerged as important tools in clinical hepatology. Measurement of HVPG is considered the gold standard for detecting clinically significant portal hypertension, with an HVPG of ≥10 mmHg being the key prognostic threshold in patients with compensated advanced chronic liver disease (cACLD; compensated cirrhosis). A transjugular liver biopsy can be obtained within the same procedure and may be preferred over percutaneous liver biopsy in patients with coagulopathy, ascites and/or significant obesity. Endoscopic ultrasound-guided procedures are currently under investigation and require standardisation. This article summarises critical technical aspects of HVPG measurements and transjugular liver biopsy and provides a detailed overview of their current role in the context of emerging non-invasive tests and endoscopic approaches.

## Graphical abstract

**Figure:**
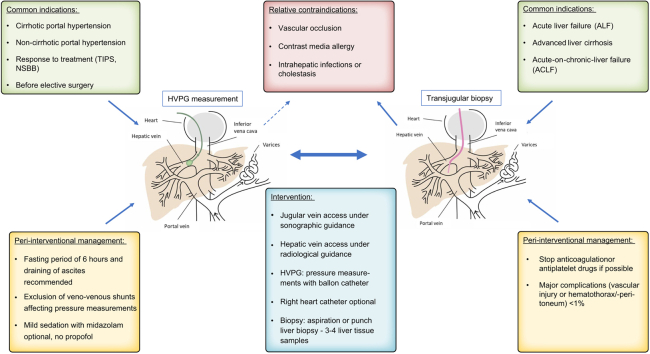

**Key points**

• Hepatic venous pressure gradient is the reference measurement for quantifying portal hypertension, which enables patient stratification, guides therapeutic management and monitoring, and predicts post-interventional liver failure and mortality.
• A standardised technique is critical to obtain accurate and reproducible results.
• Interpretation of the hepatic venous pressure gradient measurement depends on the underlying aetiology of the liver disease and may require histology for correct classification.
• The transjugular liver biopsy is a safe technique even in patients with advanced chronic liver disease or fulminant liver failure.
• Endoscopic ultrasound-guided portal pressure gradient measurement and liver biopsy represent innovative approaches, however, more evidence and standardisation are needed.

## Introduction

Interventional transjugular procedures play an increasingly relevant role in the diagnosis of acute and chronic liver diseases. Measurement of the hepatic venous pressure gradient (HVPG) has become a relevant tool in clinical hepatology as it is considered the gold standard for diagnosing sinusoidal portal hypertension in patients with compensated advanced chronic liver disease (cACLD; compensated cirrhosis) according to the current Baveno VII consensus. HVPG measurement can also be combined with transjugular liver biopsy (TJLB). Performing the two procedures together enables haemodynamic data to be correlated with underlying histopathological changes, thus facilitating a more comprehensive understanding of the pathophysiology of the underlying liver disease. However, non-invasive tests (NITs) and more recently endoscopic procedures such as endoscopic ultrasound (EUS) have emerged as relevant tools for the detection of clinically significant portal hypertension (CSPH), challenging the regular use of HVPG measurement in clinical practice. The aim of this consensus document of the *German (D) – Austrian (A) – Swiss (CH) portal hypertension (DACH-PH) consortium* is to summarise clinical and technical aspects of HVPG measurement and transjugular liver biopsy, promote standardisation and outline their use in clinical practice (Table 1). We also highlight current controversies and the implications of emerging NITs and endoscopic approaches on the use of transjugular approaches.

**Table 1:** Standardised protocol including quality measures for HVPG measurement and TJLB.

Step-by-step protocol	Quality measure
**Preparations**	
• Basic equipment: • Digital x-ray fluoroscopy system • Pressure recorder and transducer connected to printer or recording software • Ultrasound device • Sterile working area	• Quiet and sterile working area • Room approved for procedures using x-rays • Personnel trained and familiar with procedure/equipment
Pressure measurement: • Calibrate the pressure transducer • Place the transducer at the level of the patient’s right atrium (mid-axillary line) • Prepare paper or digital recording (scale range 0-50 mmHg, low recording speed 1-7.5 mm/s)	• Use pre-calibrated transducer or external pressure reference • Print calibration scale and level the zero
Patient: • Ensure patient is fastened and has consented to the procedure • Use no sedation or low doses of midazolam (0.02 mg/kg) • Prepare sterile conditions (disinfection, sterile equipment and covers)	• Written informed consent including potential risks • Monitor patient’s vital signs (blood pressure, oxygen saturation, ECG)

**Venous access and placement of the catheter**	
Central venous access: • Choose the most adequate access route (usually right internal jugular vein) • Insert a vascular access sheath (9 F for HVPG measurement and liver biopsy) using local anaesthetic and the Seldinger technique	• Choose and gain vascular access using ultrasound guidance • Fluoroscopically check and document correct placement of sheath and guidewires
Hepatic vein access: • Use balloon-tipped catheters of 10-12 mm balloon diameter • Flush and advance catheter under fluoroscopic guidance via the right atrium and the IVC into a (usually right) hepatic vein • Ensure a stable position of the catheter with the inflated balloon 1-3 cm distant to the IVC adequately occluding the hepatic vein	• Check for correct position and adequate occlusion of the vein (wedge position) using contrast agent injection into the distal hepatic vein • Observe stasis of the contrast media and exclude wash-out via vein-vein communications • If there is no stable position within the hepatic vein a rigid catheter may be used.

**Haemodynamic measurement**	
Recordings: • RA/IVC: measure pressures in the RA and IVC near the ostium of the hepatic vein • FHVP: measure pressure 2-3 cm from the hepatic vein outlet for at least 30 s or until stability occurs • WHVP: inflate balloon and measure pressure only with complete occlusion of the hepatic vein for at least 60 s or until stability occurs • Take all measurements in triplicate and calculate HVPG (FHVP subtracted from WHVP) as the mean of the three measurements	• Rinse catheter before measurements • Record measurements only when stable without variations (free and wedged pressures waveform), with the patient breathing smoothly • Repeat recording in case of artefacts such as moving or coughing • Re-evaluate recording in case of discrepancies ≥2 mmHg between measurements

**Liver biopsy**	
Transjugular access: • Choose appropriate biopsy system (aspiration biopsies usually more fragmented than core biopsies in cirrhosis) • Additional sedation or pain relief may be given once hemodynamic assessment has been completed • Insert stiff guidewire followed by the needle introducer sheath for correct position within a hepatic vein • Advance biopsy needle until the tip reaches the end of the sheath avoiding any force • Advance needle at an angle into the liver parenchyma and take biopsy • Repeat procedure until sufficient liver specimens are obtained	• Fluoroscopically check and document correct placement of sheath and guidewires • Check for correct position using contrast agent injection into the hepatic vein • Adequate biopsies are 1.5-2.5 cm in length containing 6-11 portal tracts • Take additional biopsies if specimens are less than 1.5 cm in length or fragmented • Exclude perforation of the liver capsule using contrast agent injection into the hepatic vein

**Follow-up**	
Patient: • Remove all sheaths from the vascular system and apply pressure to the access site for 5 min • Instruct patient to remain in recumbent position for the duration of the post-procedure monitoring • Administer additional pain relief or crystalloids for circulatory support as needed • Abdominal ultrasound and/or blood count check before discharge may be used to exclude post-procedure bleeding • Patients may be discharged on the day or after overnight hospital stay according to local guidelines	• Monitor patient’s vital signs (Pulse, blood pressure, oxygen saturation) for 3-8 h according to local guidelines • Discharge patient only if hemodynamically stable without evidence of bleeding, pain or shortness of breath • Discharge patient with oral and written advice on activities (driving/sport) for 24-48 h and behaviour in case of symptoms
Biopsies: • Tissue cores should be fixated in formalin or processed as fresh or frozen samples according to local guidelines	• The pathologist should receive information on the indication of the biopsy, handling of the specimen and all relevant clinical results including previous biopsies

ECG, electrocardiogram; FHVP, free hepatic vein pressure; HVPG, hepatic venous pressure gradient; IVC, inferior vena cava; RA, right atrium; TJLB, transjugular liver biopsy; WHVP, wedged hepatic vein pressure.

## Methods

The *DACH-PH consortium* comprises a multidisciplinary committee of experts within the three countries, whose members are primarily involved in the management of liver disease and portal hypertension. The methodology used for developing the consensus document followed the procedure outlined for position statements by the EASL Governing Board.^1^ As a first step, the committee identified the main areas in the field requiring discussion. In a second step, relevant clinical questions within each area were formulated, circulated among and jointly approved by the panel. The questions were then assigned to the panel members divided into five task groups based on their individual expertise. Answers were based on a thorough review of the literature and circulated first within the group and then among all the panel for review and discussion. The final consensus document was approved by all members unanimously and is based on the best evidence available at the time of writing. As this was not a formal clinical practice guideline, panellists were responsible for making an unbiased selection of the literature without a formal Delphi process.

## Clinical and technical aspects of HVPG measurement

**Fig. 1:**
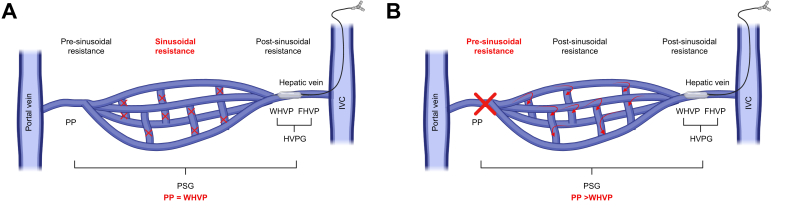
Concept of HVPG measurement. Sinusoidal portal hypertension, as typically seen in patients with viral and ALD-associated cirrhosis, is characterised by interruption of inter-sinusoidal channels. If a balloon catheter is wedged in the hepatic vein, a static pressure column is transmitted into the portal vein. As the inter-sinusoidal channels are closed, the pressure cannot be buffered. Therefore, WHVP equilibrates the PP and HVPG adequately depicts the PSG (A). In patients with non-cirrhotic portal hypertension or with increased pre-sinusoidal resistance, the inter-sinusoidal channels are open and can buffer the static pressure column. In this situation the WHVP underestimates the PP (B). Adapted from.^116^ Created with biorender.com. FHVP, free hepatic vein pressure, IVC, inferior vena cava; PP, portal pressure; PSG, portosystemic gradient; WHVP, wedged hepatic vein pressure.

CSPH is a major consequence of cirrhosis and considered the main driver of decompensation. Clinical decision-making depends on accurately assessing portal vein pressure. However, direct measurement of portal pressure using endovascular techniques is difficult because the portal vein is located between two capillary beds (hepatic sinusoids and the splanchnic capillaries). Like the pulmonary artery wedge pressure measurement, the portal pressure can be assessed indirectly by wedging the liver vein. The resulting hepatic venous pressure gradient (HVPG) is calculated by subtracting the free hepatic vein pressure (FHVP) from the wedged hepatic vein pressure (WHVP, Fig. 1).

### Preparation of the patient for HVPG measurement and TJLB

HVPG measurement requires a meticulous technical execution as several factors heavily influence the results and may lead to inaccurate pressure values. Several clinical and technical considerations are therefore essential for standardising measurements. Recent food intake increases portal vein flow, raising its pressure; hence, a fasting period of 4-6 h is recommended pre-intervention.^2^ Sedation has also been shown to affect portal vein pressure, although the procedure is generally well-tolerated and deep sedation is not required. If still needed, propofol should not be used due to its significant influence on HVPG. While fentanyl at a dose of 1.0 or 1.5 μg/kg appears to be safe with no effect on HVPG, it influences respiration and can thus cause artefacts that may decrease the accuracy of measurements.^3^ In contrast, mild sedation with midazolam (0.02 mg/kg) has no relevant influence on portal vein pressure or respiration^4^ and is therefore considered the medication of choice if sedation is required.^5^

A liver ultrasound before HVPG measurement and/or TJLB is recommended to assess pre-existing signs of cirrhosis, obstructive cholestasis or focal lesions such as abscesses, cysts and tumours which may affect the needle trajectory. Although the transjugular access route is best suited for patients with ascites, the associated displacement of the liver can impede catheterisation of the hepatic veins as an unfavourable angle forms between the inferior vena cava (IVC) and the hepatic veins, requiring pre- or peri-interventional large-volume paracentesis to rectify the anatomy. Massive ascites may also lead to large oscillations within the respiratory cycle and pressure values should be recorded in an end-expiratory state under theses circumstances.

As routine coagulation parameters do not correlate with bleeding risk in patients with cirrhosis, abnormal coagulation tests do not need correcting prior to HVPG measurement.^6^ However, a medical history, including the use of anticoagulants, should be recorded and attention paid to any susceptibility to bleeding. It should be noted that there are no absolute contraindications to transjugular interventions. Relative contraindications may be present with appropriate solutions depicted in Table 2.

**Table 2:** Summary of relative contraindications for transjugular interventions.

Relative contraindication	Possible solution
Contrast agent allergy	Premedication: • Oral premedication: methylprednisolone 32 mg at 12 and 2 h prior to contrast administration and diphenhydramine 50 mg oral, IM, or IV 1 h prior to contrast administration. • Intravenous premedication: hydrocortisone 200 mg IV 5 h and 1 h prior to contrast administration and diphenhydramine 50 mg IV 1 h prior to contrast administration.
Thrombosis of the internal jugular vein or the superior vena cava, so that the classic transjugular approach is blocked	Femoral or antecubital vascular access only if right and left internal jugular veins are occluded.
Severe coagulopathy	Puncture of the internal jugular vein should be performed under sonographic control. No routine correction of coagulation is recommended.
Anticoagulation and anti-platelet therapy	TJLB is the method of choice if liver biopsy is necessary and anticoagulation or anti-platelet therapy cannot be stopped. In case of elective procedures and the risk for omitting anticoagulation and anti-platelet therapy is low, the following recommendations should be applied: • Anticoagulation should be stopped if possible in line with the recommendations for liver biopsy.^115^ Direct oral anticoagulants should be omitted two days before the procedure (in case of renal dysfunction a longer period may be necessary). In case of warfarin a normalised INR should be achieved. • Clopidogrel, prasugrel and ticagrelor should be stopped 5-7 days before the procedure if possible. • Dual anti-platelet therapy: it should be considered if biopsy can be delayed or if clopidogrel, prasugrel or ticagrelor can be stopped. Aspirin can be continued. • Aspirin monotherapy can be continued. Anticoagulation and anti-platelet therapy can be restarted the day after the procedure.
Arrhythmias	Use of a standard guide wire (180 cm) to reach the inferior vena cava directly during jugular vein puncture in order to avoid touching the heart walls with the catheter.
Infection of the puncture site of the internal jugular vein	Contralateral access can be used.
Echinococcal cysts, liver abscesses, cholangitis, pronounced mechanical cholestasis (significant risk of infectious complications) (**only relevant for TJLB**)	Identification by periinterventional ultrasound, drainage of abscesses, bile duct stenting.
Anxious/non-compliant patient	Mild sedation with midazolam (0.02 mg/kg).

INR, international normalised ratio; TJLB, transjugular liver biopsy.

### Interventional technique of HVPG measurement and TJLB

Puncture of the right internal jugular vein is the most frequent approach to achieve vascular access for HVPG measurement. Using the left internal jugular vein is also possible, but a subsequent transjugular biopsy may be more difficult with a left-sided approach. In addition, access via the subclavian, femoral^7^ or cubital veins is possible, but such approaches are less common^8^ and rarely utilised for TJLB because of increased technical difficulties and a higher risk of complications. HVPG measurements should be performed using a balloon catheter (compliant balloons with a diameter of approximately 10-12 mm), while an end-hole catheter is generally not recommended.^9^ Employing a specifically developed and licensed, pre-shaped one-step balloon catheter not only reduces material consumption but also radiation exposure.^10^ If administering intravenous propranolol or performing right heart catheterisation as part of the intervention, the right heart catheterisation should be performed first, followed by HVPG measurement and then intravenous propranolol. The TJLB should always be left for the end of the examination, as obtaining the biopsy may require further analgesia which may affect portal pressure.

Cannulation of the hepatic vein may be more difficult in patients following orthotopic liver transplantation (OLT), especially after using a piggy-back technique for transplantation.^11^ The allograft suprahepatic caval cuff is anastomosed to the recipient’s hepatic veins rendering it more difficult to enter the veins (Fig. S1). It is highly recommended to perform HVPG measurement and TJLB in centres with experienced interventional hepatologists or radiologists, wherein technical success rates of up to 89% have been reported.^12^

**Fig. 2:**
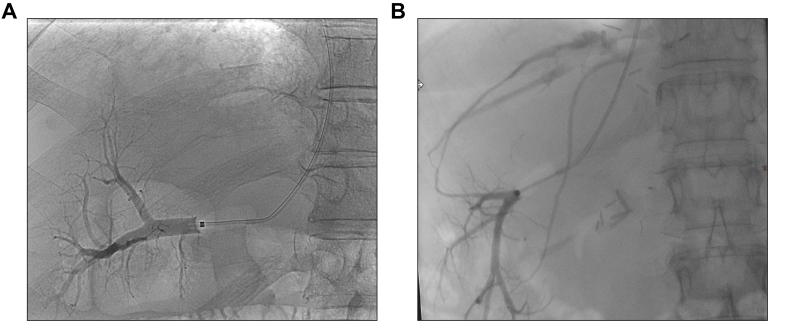
Venography during HVPG measurement. (A) Confirmation of the wedge (occlusion) position in the right hepatic vein with the balloon inflated by injecting contrast medium. (B) Veno-venous communication vessels. HVPG, hepatic venous pressure gradient.

Once the hepatic vein has been cannulated, it should be visualised by injecting a bolus of contrast medium with the balloon in a wedged position (Fig. 2A). In addition to the patency of the hepatic vein and possible obstacles to hepatic outflow, attention should be paid to the presence of veno-venous collaterals that can serve as a (non-specific) sign of porto-sinusoidal vascular liver disease (PSVD),^13^ although collaterals may also occur in patients with ACLD. The interpretation of the HVPG measurement requires careful analysis of the veno- and parenchymography, since communications between hepatic veins preclude their adequate occlusion whilst measuring WHVP (Fig. 2B). If such communications are present, the measurement should be taken more distally or in a different vessel. Ultimately, the wedged pressure may be recorded by occlusion with the catheter tip replacing the balloon; however, this approach is not as accurate and thus not routinely recommended.^14^ Notably, a FHVP measurement taken peripherally in the vein may be significantly higher than a measurement taken centrally close to the IVC, resulting in underestimation of the HVPG. Therefore, a “retracted” FHVP (FHVP ret.; max. 2-3 cm from the confluence with the IVC) is better for calculating the HVPG, if the WHVP is measured far peripherally.

**Fig. 3:**
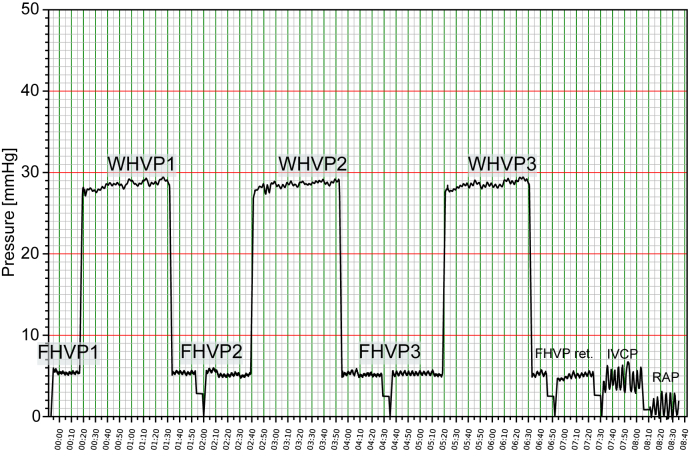
Pressure recording of FHVP, WHVP, FHVP ret., IVC pressure, and RAP. Pressure recording of the FHVP (FHVP1-3), WHVP (WHVP1-3), the optional retracted FHVP (max. 2-3 cm from the junction with the IVC; FHVP ret.), the IVCP and RAP. The “retracted” FHVP is better for calculating the hepatic venous pressure gradient, if the WHVP is measured far peripherally. FHVP, free hepatic vein pressure; FHVP ret., retracted FHVP; IVC, inferior vena cava; IVCP, IVC pressure; RAP, right arterial pressure; WHVP, wedged hepatic vein pressure.

After placing the catheter, the balloon is inflated with 1-2 ml air, but without excessive pressure as this may cause pain.^15^ The equipment should be thoroughly rinsed with saline before any measurement is taken. Once the transducer is positioned at the level of the mid-axillary line and calibrated to zero, the haemodynamic readings shown in Fig. 3 are recorded. A slow recording speed (maximum up to 7.5 mm/s) should be set and FHVP and WHVP should be measured when stability has been achieved.^5^ After pressure values stabilise, the FHVP and FHVP ret. should be measured for at least 30 s and the WHVP for at least 60 s. All measurements should be taken in triplicate. In case of significant inconsistencies (≥2 mmHg) between individual readings, technical sources of error (*e.g.* incorrect wedge position, catheter tip adjacent to vessel wall, recalibration to zero, transducer at mid-axillary level) should be excluded and the recording repeated.^15^

**Fig. 4:**
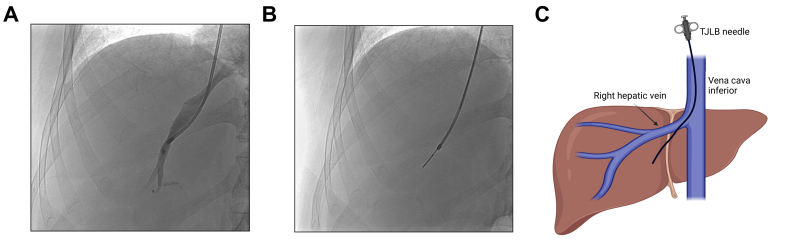
Positioning of the catheter in the right hepatic vein followed by pushed core liver biopsy. (A,B) Confirmation of the position of the introduction catheter in the right hepatic vein by injecting contrast medium (A), followed by the pushed core liver biopsy (B). (C) A schematic description of TJLB is provided.

For TJLB, two different methods are available:^16^^,^^17^ (i) the aspiration liver biopsy that can be performed by a hollow needle and (ii) the punched core biopsy which requires a needle with a clamping and firing mechanism. Both needles are introduced via a dedicated catheter (Fig. 4). A technical protocol for standardisation of HVPG measurement and TJLB is provided in Tables 1 and S2. Quality measures for each step are included to ensure accurate HVPG assessment.

### Complications of HVPG and TJLB

Both HVPG measurement and TJLB are associated with few complications and can even be performed as outpatient or overnight inpatient procedures with a short surveillance time post intervention (6 h or shorter if performed without liver biopsy). Several meta-analyses and studies are available discussing the safety profile of TJLB. Major complications occur in approximately 1% of cases.^18^^,^^19^ These include development of an intrahepatic arteriovenous fistula, vena cava injury, pneumothorax, haemothorax, haemobilia, haemoperitoneum with superinfection, liver haematoma, or liver capsule perforation.^19^ The choice of technique (core biopsy *vs.* aspiration biopsy) has no influence on the frequency of complications (for details see supplementary data).^20^ Transient cardiac arrhythmias (due to the guide wire passing through the right atrium), local skin irritation or haematoma at the access site, or accidental puncture of the carotid artery have been described. These complications usually become evident within the first 72 h following the intervention.^19^

## Clinical application of HVPG measurement

### Diagnosis of portal hypertension

Portal hypertension is a major landmark in patients with chronic liver disease and can develop because of increased resistance within the sinusoids (sinusoidal portal hypertension as in patients with cirrhosis), post-hepatically (as seen in Budd-Chiari syndrome) or pre-sinusoidally (pre-hepatic portal hypertension). The differentiation between pathophysiological mechanisms leading to portal hypertension is crucial to correctly interpret the HVPG measurement (Table 3). In patients with cirrhosis, the intra-sinusoidal and the portal vein pressure are usually close as inter-sinusoidal channels are interrupted. However, some chronic liver diseases are associated with a predominantly pre-sinusoidal component (*i.e*. vascular and cholestatic liver diseases such as PVSD, biliary atresia and primary biliary cholangitis^7^^,^[21], [22], [23]), which is characterised by open inter-sinusoidal channels and not detected by the WHVP. Thus, HVPG readings lead to an underestimation of the portal pressure (gradient). In half of these cases veno-venous communications between hepatic veins further preclude the accurate measurement of WHVP.

**Table 3:** Summary of pressure profiles for different forms of portal hypertension.

Form of portal hypertension	Pre-hepatic	Intrahepatic			Posthepatic
		Pre-sinusoidal	Intra-sinusoidal	Post-sinusoidal	
	Portal vein thrombosis	Primary biliary cholangitis, PSVD, schistosomiasis MASLD∗	Cirrhosis (viral, alcoholic, MASLD)	Sinusoidal obstruction syndrome∗∗	Budd-Chiari syndrome (congestive hepatopathy)
Pressure in the RA	Normal	Normal	Normal	Normal	Normal or ↑
IVC	Normal	Normal	Normal	Normal	Normal or ↑
FHVP	Normal	Normal	Normal	Normal	↑
WHVP	Normal	Normal or ↑	↑	↑	↑
HVPG	Normal	Normal or ↑	↑	↑	Normal
Portal vein pressure	↑	↑	↑	↑	↑
Intrasplenic pressure	↑	↑	↑	↑	↑

FHVP, free hepatic vein pressure; HVPG, hepatic venous pressure gradient; IVC, inferior vena cava; MASLD, metabolic dysfunction-associated steatotic liver disease; RA, right atrium; WHVP, wedged hepatic vein pressure.

∗ It has been shown that hepatic decompensation occurs in up to 9% of patients with MASLD with an HVPG <10 mmHg (not the case in patients with hepatis C virus infection) indicating that these patients may have an additional pre-sinusoidal component of portal hypertension).^32^^,^^34^

∗∗ Low HVPG values do not rule out sinusoidal obstruction syndrome. Therefore, HVPG measurement should be combined with transjugular liver biopsy.^109^

### HVPG measurement and its role in assessment of disease severity and prognosis

Assessing portal pressure by HVPG measurement has emerged as the best validated method for determining the prognosis of patients with ACLD irrespective of its aetiology (Table 4). Several studies confirmed that higher HVPG values are associated with an increased risk of decompensation and mortality. In compensated cirrhosis, the risk of decompensation increases once the HVPG reaches 10 mmHg, defining the cut-off value for CSPH.^24^ If the HVPG exceeds 16 mmHg, the risk of death rises significantly[25], [26], [27] and cirrhotic portal vein thrombosis is more likely to develop.^28^ Following successful etiological treatment, HVPG can predict mortality, as shown in patients with chronic HCV infection and sustained virological response, in whom an improving HVPG directly translates into an improved prognosis. Even in more advanced stages of chronic liver disease and acute-on-chronic liver failure (ACLF), HVPG provides prognostic information.[29], [30], [31]

**Table 4:** Summary of the interpretation of the measured values in an HVPG measurement in patients with cirrhosis.

Individual measurements HVPG	Interpretation
1 to 5 mmHg	Normal value. The diagnosis of cACLD/cirrhosis should be verified.
6 to 9 mmHg	Subclinical portal hypertension
≥10 mmHg	Clinically significant portal hypertension • Increased risk of having varices • Increased risk of first hepatic decompensation • Increased risk of complications after liver resection
≥12 mmHg	Increased risk of variceal bleeding
≥16 mmHg	Increased mortality risk in all settings, including in the setting of extrahepatic surgery
≥20 mmHg	Increased risk of rebleeding, refractory bleeding, and bleeding-related mortality

**Treatment aims of NSBB therapy**	

Primary bleeding prophylaxis for high-risk varices	HVPG reduction to <12 mmHg or by >10%
Secondary prophylaxis of variceal bleeding	HVPG reduction to <12 mmHg or by >20%
Prevention of decompensation in general	HVPG reduction >10% or <10 mmHg

cACLD, compensated advanced chronic liver disease; HVPG, hepatic venous pressure gradient; NSBB, non-selective beta blocker.

As some chronic liver diseases are characterised by a pre-sinusoidal component, HVPG may not fully reflect the real increase in portal pressure. More recent data indicate that HVPG measurement may also underestimate portal pressure in some patients with metabolic dysfunction-associated steatotic liver disease (MASLD). Decompensation often occurs at lower HVPG values (reflecting a pre-sinusoidal component of portal hypertension in up to 9% of patients with MASLD) than in patients with other aetiologies,^32^^,^^33^ although HVPG and CSPH still remain strong predictors of disease progression.^34^ The same is observed in patients with PVSD where signs of clinical portal hypertension develop at lower HVPG levels. Although HVPG is associated with survival, the severity of liver disease, liver and renal function together with specific portal hypertensive complications (ascites) exhibit a more pronounced prognostic effect in multivariate models.^35^ Nevertheless, the combination of HVPG and TJLB provides relevant information in these patients as typical histological parameters are necessary for the diagnosis of PSVD and facilitate the interpretation of haemodynamic parameters, in particular if signs of portal hypertension develop at low HVPG values. Combining spleen elastography and HVPG may help determine the pre-sinusoidal component, although more data are needed to assess the relevance of combining NITs and HVPG.

In children, HVPG measurement is less well validated than in adults. Elevated values were observed in acute liver failure (ALF) (median 10 mmHg) and chronic liver disease (median 7 mmHg), while HVPG remained normal in non-cirrhotic portal hypertension. The higher median HVPG was associated with general signs of portal hypertension.^36^ However, it did not correlate with the severity of liver fibrosis or inflammation and significant differences in children with or without varices, variceal bleeding, splenomegaly or ascites were missing. As children with ACLD often suffer from biliary atresia, where veno-venous communications are common, HVPG measurements must be interpreted cautiously. While transjugular interventions are safe in children, with a complication rate of 2.4%,^37^ they are not as established for risk stratification in ACLD as in adults.

### The role of HVPG measurement in patients considered for treatment with non-selective beta blockers in comparison to NITs

The Baveno VII recommendations emphasise preventing decompensation in compensated ACLD using non-selective beta blockers (NSBBs), preferably carvedilol, due to its additional alpha1-antagonistic effect, which lowers sinusoidal resistance and HVPG more effectively than propranolol.^38^ Previously, NSBB treatment was based on clinical and endoscopic signs of high-risk varices (>5 mm, red signs, Child-Pugh C cirrhosis) and not on HVPG guidance.^39^ However, now the treatment goal is not only to prevent variceal bleeding, but any first decompensating event, including the occurrence of ascites or overt hepatic encephalopathy, which may occur despite the absence of varices.^5^ This paradigm shift is based on the PREDESCI trial demonstrating that NSBBs reduce the incidence of decompensating events by 50%, particularly in patients with small varices.^40^ A meta-analysis confirmed this benefit and an improved survival, reinforcing the need to assess all patients with compensated ACLD for CSPH.^41^ Given that assessing HVPG in all patients with compensated cirrhosis is hardly feasible, other alternatives have been explored. Thus, the current Baveno VII recommendations recognise that NITs, particularly liver stiffness measurement (LSM) via vibration-controlled transient elastography, are sufficiently accurate for detecting CSPH in everyday clinical practice.^42^^,^^43^ According to the “rule of five”, CSPH can be assumed in patients with LSM >25 kPa and NSBB treatment should be started to prevent decompensation. At lower LSM values, platelet count is additionally used for categorisation.^5^ Ruling out CSPH in patients with compensated liver disease, marginal portal hypertension below the threshold value, and a low risk of disease progression is equally important. These patients do not benefit from NSBB treatment as no reduction in clinically relevant endpoints is achieved at the cost of relevant side effects.^44^ This is likely because the effect of NSBBs depends on a hyperdynamic circulation, usually observed when the HVPG exceeds 10 mmHg (defining CSPH),^45^ highlighting the need for accurate assessment of portal hypertension to avoid unnecessary and potentially harmful interventions.

However, 40-60% of patients with cACLD fall into the diagnostic “grey zone” if the “rule of five” is applied. Additional parameters like the VITRO score, spleen stiffness measurement, or endoscopy may help to refine CSPH assessment.^46^^,^^47^ A *post hoc* analysis of the PREDESCI study showed that adding endoscopy to Baveno VII criteria significantly reduced the number of patients allocated to the grey zone. Spleen elastography was also useful and further analyses are warranted to confirm these observations.^48^

Recent studies suggest that combining NITs and endoscopy may outperform HVPG in guiding NSBB initiation.^49^ Randomised-controlled trials focusing on prevention of decompensation based on non-invasive criteria of CSPH are not available but would be desirable, as retrospective data have shown promising results. However, in general practice, liver and/or spleen elastography and/or endoscopy may not always be available. A recent observational study confirmed the protective effect of carvedilol on decompensation and liver-associated mortality compared with beta-1-selective beta blockers (atenolol, metoprolol, bisoprolol, nebivolol) in patients with compensated Child-Pugh A cirrhosis and platelet counts between 30 and 150/nl in the absence of information on liver stiffness or variceal status.^50^ In summary, the unavailability of HVPG measurement or specific NITs should not prevent clinicians from prescribing carvedilol for prevention of decompensation in patients with suspected CSPH.

While not necessarily required in everyday clinical practice, HVPG measurement is thus particularly useful for assessing the presence or absence of CSPH in patients within the grey zone as classified by LSM, in particular when results have relevant clinical implications and a precise assessment of portal hypertension is crucial (*e.g*. in the context of surgical risk assessment). Under these circumstances, HVPG measurement can be considered as the diagnostic method of choice, as NITs have not been validated and may be confounded by underlying malignant disease/hepatic tumours. In patients with ascites, HVPG measurement might be the only way to confirm or rule out portal hypertension as an underlying factor.

### Assessment of the haemodynamic response to NSBBs using HVPG measurement

Assessment of the haemodynamic response to NSBBs may be useful for individualised risk stratification. Notably, response criteria, as well as the timing and frequency of treatment response assessments, have not been standardised across studies. A haemodynamic response to NSBBs given for the prevention of the first decompensation is defined as an HVPG reduction of >10% from baseline or an absolute reduction in HVPG to <10 mmHg. In secondary prophylaxis, a >20% reduction or a decrease below 12 mmHg is associated with a reduced likelihood of variceal bleeding or decompensating events and consequently with improved transplant-free survival.[51], [52], [53] The response to NSBBs can be assessed immediately by measuring the decrease in HVPG after intravenous propranolol or by repeated interventions during oral treatment. HVPG measurements have mostly been repeated approximately 4 to 12 weeks after initiation of NSBB treatment,^51^ but also confer prognostic information 1 year after initiation of treatment. In the PREDESCI study, a reduction of the HVPG of >10% or to below 10 mmHg after 1 year of treatment was associated with a reduced risk of decompensation and death (Table 4).^40^ These data confirm the results of the study by Groszmann *et al.* showing that an HVPG reduction of >10% after 1 year of treatment with timolol resulted in a significantly lower probability of developing gastroesophageal varices or variceal bleeding.^44^

The acute response to intravenous propranolol can be assessed within the same session as the initial measurement. An acute *non-response* to intravenous propranolol (HVPG reduction <10-12%) is associated with an increased risk of variceal bleeding and development of ascites.^40^^,^^54^^,^^55^ Moreover, the acute response to intravenous propranolol, defined as a drop in HVPG of >10% or to an HVPG below 12 mmHg, is expected in about two-thirds of patients with CSPH^40^^,^^56^ and can predict long-term response to oral NSBB treatment.^56^ However, a non-response to intravenous propranolol does not preclude that a patient may respond to carvedilol^56^ and the acute haemodynamic response 60 to 90 min after oral administration of carvedilol may indeed predict long-term response, though these observations have yet to be evaluated with respect to clinically relevant endpoints.^57^^,^^58^

With personalised treatment approaches emerging in hepatology, HVPG-guided treatment with NSBBs could become an important part of clinical management. In contrast, the routine use of HVPG to guide NSBB treatment has been subject to ongoing discussion in the scientific community. Analysis of published randomised-controlled trials ^59^^,^^60^ has raised questions about the usefulness of HVPG in individual patients, particularly in those with decompensated cirrhosis. Further investigations are warranted to fully understand the implications of this finding in clinical practice. Meanwhile, the utility of NITs in assessing the haemodynamic response to NSBBs has not been established and changes in liver stiffness do not correlate well with changes in HVPG. Spleen elastography holds promise but remains to be validated^61^^,^^62^ and routine assessment of the haemodynamic response to NSBBs is currently not recommended.

### HVPG measurement before and portosystemic pressure gradient measurement during and after TIPS implantation

Transjugular intrahepatic portosystemic shunt (TIPS) is an important disease-modifying intervention for decompensated cirrhosis, with variceal bleeding and refractory or recurrent ascites being the most common indications.^63^ If ascites is not clearly due to portal hypertension, HVPG measurement should be performed before TIPS. In preemptive TIPS, an HVPG >20 mmHg within 24 h of bleeding predicts a higher rebleeding risk, though today HVPG is not routinely used as the high-risk population is defined according to clinical and endoscopic parameters (Child-Pugh class C <14 points or Child-Pugh class B >7 with active bleeding at initial endoscopy).

During TIPS implantation, direct portal vein and IVC pressures are measured to calculate the portosystemic gradient (PSG).^64^ The accurate position of the catheter for measurement of these values is essential. Indeed, use of the post-TIPS portoatrial gradient overestimates the real PSG by a mean of 2.5 mmHg compared to the portocaval gradient.^65^ This is relevant as a significant proportion of patients may still have a HVPG >12 mmHg. The main goal of TIPS implantation is pressure reduction to treat/prevent further portal hypertension-associated complications. A pressure reduction after TIPS implantation of ≥50% or to a PSG <12 mmHg prevents rebleeding, while a 60-80% reduction is associated with an increased rate of ascites clearance without increasing the incidence of post-TIPS hepatic encephalopathy.[66], [67], [68], [69]

As for HVPG measurement, deep sedation significantly affects PSG. Deep sedation or general anaesthesia during TIPS implantation results in lower PSG values immediately post-procedure, with levels increasing after 24 h in stable, unsedated conditions. A second PSG measurement 24-72 h post-TIPS without sedation correlates with clinical outcomes and should guide further management, such as stent dilation.^70^^,^^71^ Notably, patients with no PSG increase within 24 h may have worse prognoses.^72^ Taken together, a second PSG measurement in a stable, unsedated patient is recommended after TIPS implantation as it reflects accurate pressure values that are useful for prognostication and decision-making, *e.g.* further dilation of the stent to reach the anticipated PSG target.

### HVPG measurement before abdominal surgery in patients with cirrhosis

Extrahepatic surgical interventions may lead to postoperative decompensation and ACLF, which are associated with increased mortality. Typical complications include infections, acute kidney injury with or without aggravation of ascites, and ACLF.[73], [74], [75], [76], [77], [78] Common indications for elective surgery in patients with compensated cirrhosis are gallstones and abdominal wall hernias.^79^^,^^80^ Apart from the MELD (model for end-stage liver disease) and Child-Pugh scores or specifically designed models such as the VOCAL-PENN score,^81^^,^^82^ CSPH is the most important determinant of postoperative morbidity and mortality.^75^^,^^78^^,^[83], [84], [85] If present, portal hypertension can be indirectly assessed by signs such as a low platelet count, splenomegaly, varices, ascites or previous decompensation prior to surgery.^77^^,^^86^^,^^87^ The presence of CSPH can be assumed if there are typical signs (varices at endoscopy or portosystemic collaterals on CT scans); in such cases, a higher peri- and postoperative risk can be anticipated. However, in more than 50% of compensated patients with CSPH, clinical indicators of portal hypertension are missing and the platelet count is normal.^88^ Non-invasive methods can give important information regarding the absence/presence of CSPH, but HVPG measurement provides a higher degree of certainty and granularity. Reverter *et al.* were able to show that an HVPG value above 16 mmHg is an independent prognostic factor for increased postoperative mortality in patients with Child-Pugh A and B cirrhosis.^89^ Therefore, HVPG measurement is an important component of multimodal risk stratification before surgery.

Liver resection for hepatocellular carcinoma is of particular interest and HVPG measurement helps to identify patients suited for surgical resection. Since hepatic resistance increases after partial hepatectomy, a preoperative HVPG >10 mmHg is accompanied by excessive rates of decompensation and mortality.^90^ Recently, technical advances have challenged this threshold,^91^^,^^92^ making a more individualised approach desirable. Thus, further data defining new cut-offs for HVPG are needed.

Finally, OLT is a surgical procedure associated with a high risk of major bleeding events in patients with cirrhosis. HVPG before OLT can predict these events, as an HVPG >16 mmHg is associated with a high risk (and an HVPG >20 mmHg with a very high risk) of bleeding complications.^21^ Therefore, detailed information on HVPG before OLT may help surgical teams to better anticipate the perioperative course. The combination with right heart catheterisation adds further important haemodynamic data for better risk stratification. This may pave the way for the inclusion of HVPG measurement and right heart catheterisation in pre-transplant preparation, and further prospective studies should assess their use in this setting.

### Right heart catheterisation during HVPG measurement

Cirrhosis may be associated with cardiac and pulmonary abnormalities such as cirrhotic cardiomyopathy, portopulmonary hypertension or hepatopulmonary syndrome. Cardio-pulmonary comorbidities are particularly relevant in the evaluation for OLT or TIPS implantation. As HVPG measurement provides relevant information about the perioperative course, the combination of both examinations is encouraged in pre-transplant preparation.^21^ Transthoracic echocardiography (TTE) is the standard screening method in patients with suspected portopulmonary hypertension or hepatopulmonary syndrome (combined with transthoracic contrast-enhanced echocardiography for detection of intrapulmonary vascular dilatations and shunts) and is recommended in patients undergoing OLT or TIPS evaluation. TTE should also be performed in any patient with dyspnoea and/or clinical signs of cardiac decompensation.^93^ Patients with TTE-based estimation of the systolic pulmonary arterial pressure exceeding 40-50 mmHg, significant right ventricular hypertrophy or right ventricular dysfunction have to undergo invasive assessment of pulmonary haemodynamics by right heart catheterisation (recording the parameters depicted in Table S1).^93^

Right heart catheterisation can also be helpful in diagnosing hyperdynamic circulation in patients with cirrhosis by determining the cardiac output or cardiac index. A cardiac index >4.2 l/min∗m^2^ and a low systemic vascular resistance (=(mean arterial pressure – central vein pressure)/cardiac output × 80) is considered a hyperdynamic state^26^ and the basis for NSBB treatment to be effective^45^ .^57^ This – together with the ability to detect (porto)pulmonary hypertension – serves as the rationale for combining HVPG with right heart catheterisation.

### EUS-guided portal pressure measurement

During the last years, portal pressure measurement using endoscopic ultrasound (EUS) has become feasible. This emerging field of endohepatology including EUS-guided portal pressure measurement (EUS-PPG) can overcome most of the limitations associated with HVPG measurement. By directly measuring portal and hepatic vein pressures, the risk of underestimated values in the aforementioned clinical scenarios (PBC, PSVD, some MASLD patients) is eliminated. Technical success rates for EUS-PPG of 96-100% have been reported.[94], [95], [96] Preliminary data suggests a good correlation of HVPG measurement and EUS-PPG in patients with cirrhosis, as well as in patients with non-cirrhotic portal hypertension.^97^^,^^98^ However, EUS is mainly performed with propofol sedation, which may affect pressure values. A study in which HVPG was measured without sedation and EUS-PPG was measured in the same patients with propofol sedation reported mean pressure values that were actually slightly higher with EUS-PPG, highlighting the controversial effect of sedation on EUS-PPG measured values.^98^ The non-physiological conditions caused by posture and the endoscopic intervention may additionally impede accurate measurements. Finally, although assumed to be relatively safe, the risk should not be different from that of percutaneous transhepatic PPG measurement, which was the standard technique until the advent of EUS-PPG. Since most published series are small, safety data will remain a crucial aspect of future studies.

To date, EUS-guided pressure values have not been sufficiently correlated with clinical outcome parameters (decompensation, survival) (Table 4). Although the combination of EUS-PPG with EUS-guided liver biopsy has the same advantages as HVPG measurement and TJLB in combining both procedures in the same session, HVPG and TJLB are still the gold standard, as their safety and correlation with clinically relevant endpoints has been widely validated.

## TJLB in clinical practice

TJLB has emerged as an important tool in clinical hepatology when a liver biopsy is necessary but cannot be performed percutaneously. The presence of ascites in the anticipated needle trajectory is an absolute contraindication to percutaneous access because of the increased risk of uncontrollable bleeding, while TJLB remains the safest alternative.^99^^,^^100^ Laboratory evidence of coagulopathy is also considered a relevant contraindication to percutaneous biopsy, in particular in patients with concomitant ascites. In contrast, TJLB is associated with a low bleeding risk^101^ and is considered safe for patients with impaired coagulation.^19^

A major advantage of TJLB is that it can be performed during the same intervention as HVPG measurement, therefore providing additional information about the underlying liver disease. Several studies focus on the correlation of histopathological changes with the degree of portal hypertension. The extent of hepatic fibrosis (Scheuer stage) and the HVPG show a significant correlation (r = 0.654, *p* <0.001).^102^ Patients with advanced septal fibrosis can have CSPH and are thus at risk of decompensating events.^103^ Taken together, the combined use of TJLB and HVPG is recommended in a relevant work-up.

However, transjugular biopsies are generally untargeted and focal liver pathologies cannot be assessed with sufficient accuracy unless ultrasound guidance is provided in parallel by a second interventionalist (mainly for lesions in the right hepatic lobe). The most important indications for TJLB are ALF, suspected cirrhosis or PSVD, ACLF, and systemic diseases with secondary liver involvement (veno-occlusive disease, haematological diseases). The role of TJLB in some of these entities will be discussed in detail (for specific pathological issues: see the supplementary information).

During the last years, EUS-guided liver biopsy has emerged as an important tool in hepatology. Currently, there are no specific recommendations for EUS-guided liver biopsy in patients with ascites and coagulopathy. Therefore, in this special clinical situation, TJLB remains the method of choice, especially as head-to-head comparisons for the different biopsy approaches are missing.

### TJLB in patients with ALF

ALF is a life-threatening disease associated with high mortality. It is defined by acute liver injury in combination with laboratory evidence of coagulopathy (international normalised ratio [INR] >1.5) and hepatic encephalopathy in patients without pre-existing liver disease.^104^ Evaluation of the aetiology of ALF is essential as it determines prognosis and can guide specific treatment. In patients with suspected autoimmune hepatitis-associated ALF, the result of the liver biopsy is crucial to establish the indication for immunosuppression. Given the characteristic changes in coagulation parameters, TJLB is the method of choice in ALF.

### TJLB in patients with suspected cirrhosis or PSVD or haematological diseases

The most important reason for liver biopsy in patients with fibrosis or cirrhosis is to determine the underlying disease and to assess the degree of fibrosis. Before the introduction of non-invasive tools in everyday clinical practice, liver biopsy and histologic evaluation of liver tissue were essential components of the diagnostic workup to determine the grade of fibrosis.^105^ Today, non-invasive methods (*e.g.* liver elastography) have replaced biopsies in routine clinical practice.^106^ However, histological evaluation remains necessary in some liver diseases as their severity is determined by histopathological scores (*e.g*. autoimmune hepatitis,^107^ unclear cholestatic liver disease, and definite diagnosis of MASLD^108^). Liver biopsy is therefore indicated if the cause of cirrhosis is unclear and the result will change therapy.

Other rare indications include evaluation of veno-occlusive or graft *vs.* host disease after stem cell transplantation. In this setting, the combination of HVPG measurement and TJLB provides relevant information for further management as these entities may be associated with portal hypertension while the histological features help to classify haemodynamic findings.^109^

### TJLB in patients with ACLF

The definition of ACLF is exclusively based on clinical parameters (acute decompensation of cirrhosis with organ dysfunctions/failure) without considering histopathological changes. Few studies have focused on histopathological results in relation to the extent of organ failure and local inflammation.^110^ In a retrospective study, liver specimens of 152 patients with ACLF (EF-CLIF criteria) were analysed and compared to 98 samples from patients with compensated cirrhosis. Samples from patients with ACLF showed significantly more necrotic areas, cholestasis and interface hepatitis compared to those from patients with compensated cirrhosis. Similar to patients with ALF, the extent of necrotic areas was a negative predictor of survival.^111^ Liver biopsy should be considered in patients without previously known liver disease who present with rapidly progressive impairment of liver function. Apart from assessing prognosis, evaluating the presence and aetiology of cirrhosis is essential for deciding on further therapies and potentially also priority for OLT. The role of TJLB and HVPG in the assessment of patients with ACLF prior to OLT should be evaluated in further clinical studies to shed light on specific haemodynamic and histological predictors of worse outcomes.

### Contraindications to TJLB, management of coagulation, anticoagulants and anti-platelets drugs before TJLB

There are no absolute contraindications to TJLB apart from the impossibility of gaining access to the hepatic veins, a severe contrast allergy or intrahepatic infections (Table 2). TJLB is associated with a low bleeding risk.^6^ Markers of haemostasis (INR, platelet count, fibrinogen) are frequently altered in patients with cirrhosis. However, as pro- and anticoagulation parameters are reduced, a status of rebalanced haemostasis can be assumed.^112^ Therefore, substitution of coagulation products is not necessary. There are no studies that define thresholds for the indication of blood products prior to TJLB. Sue *et al.* analysed the complication frequency of 1,321 TJLB procedures in 932 patients. The rate of complications or bleeding events was not increased in patients with platelet counts <50 x 10^9^/L compared to patients with higher platelet counts. Even in patients with an INR >3.0, bleeding was not increased.^19^ These data were reproduced by Lee *et al.* in 603 patients after OLT who underwent TJLB.^113^ The bleeding rate in the overall cohort was 1.0%. Appropriate substitution is, however, recommended for patients with hereditary coagulation disorders.^114^

One important issue to be considered is the use of anti-platelet drugs and/or anticoagulation. As TJLB is defined as an intervention with a low bleeding risk, anti-platelet drugs could theoretically be continued; however, in everyday clinical practice, anticoagulation is stopped for the intervention and can be restarted the day after the procedure.

## Conclusion

Transjugular interventions in hepatology provide relevant information for clinical practice and are still superior to other approaches in terms of accuracy and safety. In particular, the combination of HVPG measurement and TJLB facilitates patient management, increases the diagnostic yield and guides personalised treatment decisions in a number of clinical contexts. Current indications include the assessment of CSPH, if NITs and endoscopy are not available or are inconclusive. The detection of CSPH in combination with pulmonary and systemic haemodynamics during right heart catheterisation helps to determine the reason for ascites if it is not clearly linked to portal hypertension. HVPG measurement plays an important role in assessing CSPH before TIPS implantation while its use before surgical procedures should be extended. In clinical trials, HVPG is an essential parameter to evaluate the response to specific treatments. The combination with TJLB provides further histological insights that are useful for understanding chronic liver diseases and allows biopsies in patients with A(C)LF with ascites and/or increased bleeding risk.

Transjugular interventions are often performed by interventional radiologists who are not directly involved in the management of patients with ACLD. Close cooperation and, whenever possible, the joint implementation of these interventions ensures appropriate standardisation and thus adequate results, which are crucial to guide subsequent patient management. As these interventions represent a major component in the diagnostic armamentarium, hepatologists should be encouraged to perform these procedures themselves and obtain expertise as interventional hepatologists.

## Abbreviations

ACLF, acute-on-chronic liver failure; ALF, acute liver failure; cACLD, compensated advanced chronic liver disease; CSPH, clinically significant portal hypertension; CT, computer tomography; FHVP, free hepatic venous pressure; HE, hepatic encephalopathy; HVPG, hepatic venous pressure gradient; INR, international normalised ratio; IVC, inferior vena cava; LSM, liver stiffness measurement; MASLD, metabolic dysfunction-associated steatotic liver disease. MELD, model of end stage liver disease; NIT, non-invasive tests; NSBB, non-selective betablocker; OLT, orthotopic liver transplantation; PSG, portosystemic pressure gradient; PVSD, porto-sinuosoidal vascular liver disorder; TIPS, transjugular intrahepatic portosystemic shunt; TJLB, transjugular liver biopsy, TTE, transthoracic echocardiography; WHVP, wedged hepatic venous pressure.

## Financial support

The authors did not receive any financial support to produce this manuscript.

## Authors’ contributions

Coordination: DB, MD. Draft of the manuscript: DB, AB, CL, EZ, MM, CR, VF, MP, TB, CJ, CM, AZ, RM, AdG, AF, BM, CE, BM, JE, MS, PR, KL, JH. Critical revision of the manuscript: DB, TR, JT, RK, AT, PS, CML, CS, MD, JB.

## Conflicts of interest

DB: Lecture fees/consulting: W. L. Gore & Associates GmbH, Travel grant: Gilead Science.

AB: Consulting: Boehringer-Ingelheim; lecture fees: W. L. Gore & Associates GmbH; GE Healthcare; Hologic. MM: Speaker and/or consultant and/or advisory board member for AbbVie, Collective Acumen, Gilead, Echosens, Ipsen, Takeda, and W. L. Gore & Associates; travel support from AbbVie and Gilead. CR: Consulting: Boehring-Ingelheim, lecture fees: W. L. Gore & Associates GmbH, Falk Foundation, Bristol-Myers Squibb. EZ: lecture fees: Abbvie, Gilead, Dr. Falk Pharma; travel grants: Abbvie, Gilead, W.L. Gore& Associates; advisory: Bentley InnoMed. TB: received consulting fees from Intercept/Advanz Pharma, Grifols, and Sobi; honoraria for lectures, presentations, or educational events from Falk Foundation, CSL Behring, Merck, Gilead, Intercept/Advanz Pharma, and Gore; travel support from Gilead. CE: Advisory: Boehringer-Ingelheim, Albireo, Lecture fees: Albireo, Gilead. VF: Advisory: Astra Zeneca, ADVITOS, Lecture fees: CSL-Behring, ADVITOS, Astra Zeneca, Merz. PAR: lecture fees: Pfizer, BMS, CSL Behring, CSL Seqirus, AstraZeneca; Advisory Board: Gilead, ADVANZ, Pfizer, travel support AbbVie, Ipsen. MS: lecture fees/consulting: Falk Foundation e.V., W. L. Gore & Associates, Bentley InnoMed GmbH. AZ: Lecture fees/consulting: CLS Behringer, Lecture fees: W. L. Gore & Associates GmbH, Falk Foundation, CML: consulting fees from Abbvie, AstraZeneca, Boston Scientific, CSL Behring, Eisai, Falk, Gilead, Norgine, Roche, Shionogi, and Sobi; honoraria for lectures, presentations, or educational events from Abbvie, AstraZeneca, Boston Scientific, CSL Behring, Gore, Eisai, Falk, and Norgine. TR: received grant support from Abbvie, Boehringer Ingelheim, Gilead, Intercept/Advanz Pharma, MSD, Myr Pharmaceuticals, Philips Healthcare, Pliant, Siemens and W. L. Gore & Associates; speaking honoraria from Abbvie, Gilead, Intercept/Advanz Pharma, Roche, MSD, W. L. Gore & Associates; consulting/advisory board fee from Abbvie, Astra Zeneca, Bayer, Boehringer Ingelheim, Gilead, Intercept/Advanz Pharma, MSD, Resolution Therapeutics, Siemens; and travel support from Abbvie, Boehringer Ingelheim, Dr. Falk Pharma, Gilead and Roche. RK: received consulting fees from Boston Scientific, Bristol Myers Squibb, Guerbet, Roche, and Sirtex and lecture fees from Astra Zeneca, BTG, Eisai, Guerbet, Ipsen, Roche, Siemens, Sirtex, MSD Sharp & Dohme and is on the data safety monitoring board of the ABC HCC Trial. Furthermore, he serves as the Chair of the Audit and Standards Subcommittee of the European Society of Radiology. All of these roles are not related to this project. JB: Consultant to AstraZeneca, Boehringer Ingelheim, Novo Nordisk and Resolution Therapeutics. MD: Lecture fees/consulting: Sanofi-Aventis, Ipsen Pharma, Allpha Sigma Pharma, Takeda Pharmaceuticals, Falk Foundation, Mainz Biomed GmbH; Travel grant: Gilead Science, Falk Foundation.

Please refer to the accompanying ICMJE disclosure forms for further details.
