# Supplementary material for: Transjugular diagnostic procedures in hepatology: Indications, techniques and interpretation
Source: JHEP Rep. 2025 Apr 29;7(8):101437. doi: 10.1016/j.jhepr.2025.101437 (PMC12269627; doi:10.1016/j.jhepr.2025.101437)
Supplement: Multimedia component 1 [file mmc1.pdf]

Transjugular diagnostic procedures in hepatology: Indications, techniques and interpretation

Dominik Bettinger, Annalisa Berzigotti, Matthias Mandorfer, Cristina Ripoll, Christian Labenz, Eugen Zizer, Tony Bruns, Andrea de Gottardi, Johannes Emrich, Cornelius Engelmann, Arnulf Ferlitsch, Valentin Fuhrmann, Benjamin Maasoumy, Jan Hinrichs, Christian Jansen, Karoline Lackner, Benjamin Maasoumy, Robert Matzberger, Carsten Meyer, Behrang Mozayani, Michael Praktikjno, Philipp Reuken, Michael Schultheiß, Alexander Zipprich, Alexander Gerbes, Christian Lange, Roman Kloeckner, Christoph Sarrazin, Jonel Trebicka, Thomas Reiberger, Jaume Bosch, Matthias Dollinger *on behalf of the German (D) – Austrian (A) – Swiss (CH) portal hypertension (DACH-PH) consortium*

Table of contents

Table S1 ……………………………………………………………………………….2

Table S2…………………………………………………………………………………3

Sample quality of TJLB in comparison to percutaneous liver biopsy………………….5

Fig. S1…………………………………………………………………………………..7

Supplementary references…………………………………………………………….8

Table S1: Summary of parameters that should be recorded during a right heart catheterization.

Measured parameters	Normal value
Mean right atrial pressure (RAP)	2-6 mmHg
Pulmonary artery pressure, systolic (sPAP)	15-30 mmHg
Pulmonary artery pressure, diastolic (dPAP)	4-12 mmHg
Mean pulmonary artery pressure (mPAP)	8-20 mmHg
Pulmonary arterial wedge pressure (PAWP)	≤15 mmHg
Cardiac output (CO)	4-8 L/min
Mixed venous oxygen saturation (SvO2)	65-80%
Arterial oxygen saturation (SaO2)	95-100%
Systemic blood pressure	120/80 mmHg
**Calculated parameters**	**Normal value**
Pulmonary vascular resistance (PVR)	0.3-2.0 Wood Units (WU)
Pulmonary vascular resistance index (PVRI)	3-3.5 WU x m2
Total pulmonary resistance (TPR)	3 WU
Cardiac Index (CI)	2.5-4.0 L/min x m2
Stroke volume (SV)	60-100ml
Stroke Volume Index (SVI)	33-47 mL/m2
Pulmonary arterial compliance (PAC)	2.3 mL/mmHg

Table S2: Standardized report of a HVPG measurement

**Demographic data**	• Name<br>• Date of birth<br>• Height, Body weight<br>• calculated BMI
**Medical history**	• Diagnosis<br>• Etiology<br>• Relevant previous operations (transjugular intrahepatic portosystemic shunt, partial liver resection, liver transplantation)
**Indication**	
**Medication**	• Beta blocker, statins, nitrates, renin- angiotensin blockers, anticoagulation, diuertics: yes/no,<br>If yes: Date/time of last administration, dose
**Sedation**	before/during the examination, medication including cumulative doses, for midazolam best body weight adapted
**Technical data**	• Access route (e.g. internal jugular vein, right or left)<br>• Size of introducer sheet (e.g. 7.5F-9F)<br>• Types of catheter(s) used, if applicable
**Pressure Measurements**	• Mean arterial pressure (MAP)<br>• Heart rate (HR)<br>• Hepatic vein pressure (WHVP; measured three times; report median value; report max 1)<br>• Free hepatic venous pressure (FHVP; measured three times; report median value)<br>• HVPG (difference from median WHVP to median FHVP; in mmHg)<br>• Pressure in the inferior vena cava (VCI)<br>• Pressure in the right atrium (RAP)
**Comments**	• Vein-to-vein communications, preventing a sufficient wedge position (yes/no)<br>• stable tracing of WHVP (yes/no)

	• Administration of medication (beta-blocker bolus)
**Transjugular liver biopsy (TJLB)**	• What type of needle (trucut, aspiration)<br>• Description of the quantity of material extracted (length, number of cylinders)
**Interpretation**	• Normal findings<br>• portal hypertension<br>• clinically significant portal hypertension<br>• Information on the response to acute medication (e.g. i.v. propranolol or study medication) when assessed by multiple measurements.
**Radiation**	• Screening time<br>• Radiation dose

Sample quality of TJLB in comparison to percutaneous liver biopsy

Sample quality plays an important role in the histological interpretation of a liver biopsy. The histological changes of most diffuse liver diseases show a more or less pronounced focal appearance in the liver tissue. Morphological characteristics are also distributed differently within a liver lobule. The biopsy should therefore contain a sufficiently large number of portal fields and central veins in a lobule to enable a representative histological assessment. This is particularly important in the assessment of liver fibrosis ("staging") and/or diseases that affect the liver in a very focal manner (e.g. chronic biliary diseases) (1–3) According to the guidelines of the American Association for the Study of the Liver (AASLD), a liver biopsy cylinder for transcutaneous biopsies should ideally be obtained with a 16 Gauge (G) needle, be 3 cm long and approximately 1 mm in diameter (4). As a minimum requirement for representative quality, a liver biopsy should be at least 20 mm long and contain more than 10 portal fields. Biopsies less than 1 cm in length and less than 6 portal fields are not representative, while biopsies with a length of 11-19 mm and between 6 and 10 portal fields are considered to be of limited representativeness (1,5). To achieve these quality criteria, it is recommended to take two biopsies from the liver. Taking a second biopsy is not associated with a significantly higher incidence of complications (6).

Compared to transcutaneous biopsy, transjugular liver biopsies are usually performed with thinner biopsy needles, usually 18G or 19G. Kalambokis et al. performed a systematic review comparing a Menghini needle (aspiration biopsy) with Tru-cut needle including 64 case series with 7649 TJLBs. Patients with parenchymal liver disease as well as patients with acute and chronic liver diseases as well as bone marrow transplant recipients and heart transplant candidates. Menghini needle specimens were more often fragmented. Significantly longer specimens were obtained using using Tru-Cut needles (median 14.5 mm) than Menghini needles (median: 9.5 mm). It has also been shown that using thinner needles (> 16 G, ususally 18 or 19 G) can provide longer specimens mainly due to reduced fragmentation. Cholongitas et al have shown that TJLB with three passes using a 19G Tru-Cut needle results in specimens with a mean length of 22.5 mm with a mean number of 8.7 complete portal tracts (7).

To ensure the representativeness of the biopsy material, several biopsies should be taken (5,7). In a recent study, representative liver tissue was obtained significantly more often with 4 biopsies than with 3 biopsies with no increase of complications. In contrast, the quality of the tissue samples taken transjugularly was not dependent on the technique used (punch biopsy vs. aspiration biopsy) (8). Due to the smaller thickness of the punch cylinders, some studies have observed in average a lower number of portal fields in the liver biopsies obtained transjugularly compared with transcutaneous or mini-laparoscopy-assisted biopsies (9).

However, this is not associated with a reduction in the ability to assess the etiology of liver disease or the severity of fibrosis.

For most questions, biopsies should be sent for histopathological evaluation completely covered in buffered formaldehyde in suitable sample containers. If Wilson's disease is suspected, a sample in formaldehyde can be sent to a chemical laboratory for histological examination and another sample unfixed (without the addition of formaldehyde or other solutions), possibly rolled up in moistened paper, for determination of liver copper. The same procedure can be used in patients with suspected primary hemochromatosis and liver iron determination. If fatty liver of pregnancy is suspected, a frozen section examination is helpful, as typical histological features (including microvesicular steatosis) may no longer be detectable after formaldehyde fixation and paraffinization. If an examination with an electron microscope is necessary (in some pediatric cases), a punch cylinder can be fixed in glutaraldehyde.

Sample quality and complications of TJLB

Kalambokis et al. also reported complications of TJLB. Smaller needles were associated with less complications and using Tru-Cut needles resulted in fewer complication rates. However, the main predictors of complications were age (especially pediatric patients) and the experience of the center (7). Importantly, it has to be mentioned that the complication rate was very low (major complications: 0.5%). Major complications may predominantly occur in patients with smaller livers and/or horizontal liver veins so that technical difficulties may occur leading to a higher perforation rate. It has been described that minor complications were more common in TJLB using Menghini needles probably due to the difficulty in controlling the depth of puncture. However, fluoroscopic control can significantly reduce this problem. As smaller Tru-Cut needles also lead to higher sample quality and fewer complications, these needles can be preferred.

Fig. S1: Allograft suprahepatic cuff after othotopic liver transplantation (OLT)

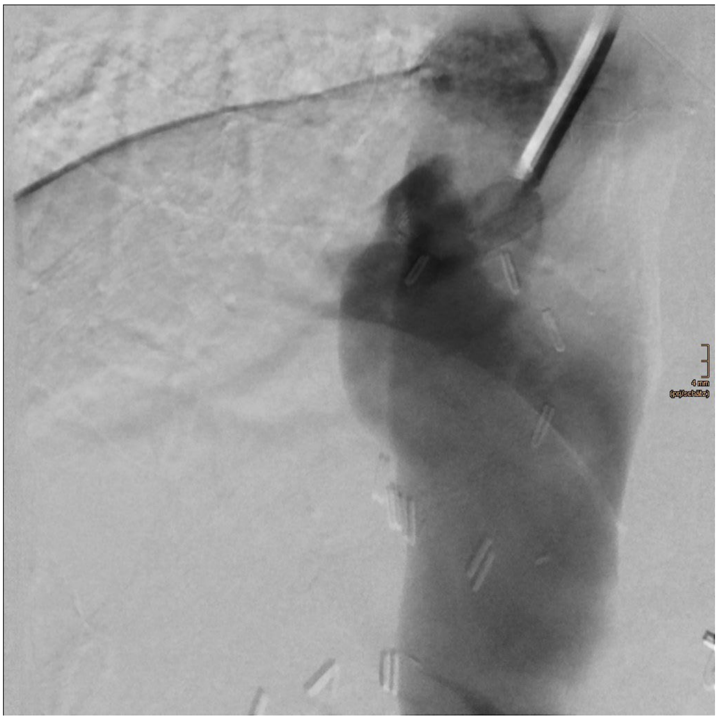

Fig. S1: Example of a patient with prior OLT performed in piggy-back technique. The angiographic picture shows the suprahepatic caval cuff that is associated with a more difficult canulation of the hepatic veins.

Supplementary references

1. Neuberger J, Patel J, Caldwell H, Davies S, Hebditch V, Hollywood C, u. a. Guidelines on the use of liver biopsy in clinical practice from the British Society of Gastroenterology, the Royal College of Radiologists and the Royal College of Pathology. Gut. August 2020;69(8):1382–403.

2. Scheuer PJ. Liver biopsy size matters in chronic hepatitis: bigger is better. Hepatol Baltim Md. Dezember 2003;38(6):1356–8.

3. Garrido MC, Hubscher SG. Accuracy of staging in primary biliary cirrhosis. J Clin Pathol. Juli 1996;49(7):556–9.

4. Rockey DC, Caldwell SH, Goodman ZD, Nelson RC, Smith AD, American Association for the Study of Liver Diseases. Liver biopsy. Hepatol Baltim Md. März 2009;49(3):1017–44.

5. Fryer E, Wang LM, Verrill C, Fleming K. How often do our liver core biopsies reach current definitions of adequacy? J Clin Pathol. Dezember 2013;66(12):1087–9.

6. Chi H, Hansen BE, Tang WY, Schouten JNL, Sprengers D, Taimr P, u. a. Multiple biopsy passes and the risk of complications of percutaneous liver biopsy. Eur J Gastroenterol Hepatol. Januar 2017;29(1):36–41.

7. Kalambokis G, Manousou P, Vibhakorn S, Marelli L, Cholongitas E, Senzolo M, u. a. Transjugular liver biopsy--indications, adequacy, quality of specimens, and complications--a systematic review. J Hepatol. August 2007;47(2):284–94.

8. Krupski G, Buggisch P, Koops A, Adam G. [Transjugular liver biopsy: trucut or aspiration biopsy with modified Ross needles?]. ROFO Fortschr Geb Rontgenstr Nuklearmed. Februar 2003;175(2):258–61.

9. Beckmann MG, Bahr MJ, Hadem J, Bredt M, Wedemeyer H, Schneider AS, u. a. Clinical relevance of transjugular liver biopsy in comparison with percutaneous and laparoscopic liver biopsy. Gastroenterol Res Pract. 2009;2009:947014.
